# Chemokine receptor hetero-oligomers regulate monocyte chemotaxis

**DOI:** 10.26508/lsa.202402657

**Published:** 2024-05-23

**Authors:** Garrett A Enten, Xianlong Gao, Michelle Y McGee, McWayne Weche, Matthias Majetschak

**Affiliations:** 1 Department of Surgery, University of South Florida , Morsani College of Medicine, Tampa, FL, USA; 2 Department of Molecular Pharmacology and Physiology, University of South Florida , Morsani College of Medicine, Tampa, FL, USA

## Abstract

It is known that stress influences immune cell function. The underlying molecular mechanisms are unclear. We recently reported that many chemokine receptors (CRs) heteromerize with α_1_-adrenoceptors (α_1_-ARs) through which CRs are regulated. Here, we show that arginine vasopressin receptor 1A (AVPR1A) heteromerizes with all human CRs, except chemokine (C-X-C motif) receptor (CXCR)1, in recombinant systems and that such heteromers are detectable in THP-1 cells and human monocytes. We demonstrate that ligand-free AVPR1A differentially regulates the efficacy of CR partners to mediate chemotaxis and that AVPR1A ligands disrupt AVPR1A:CR heteromers, which enhances chemokine (C-C motif) receptor (CCR)1-mediated chemotaxis and inhibits CCR2-, CCR8-, and CXCR4-mediated chemotaxis. Using bioluminescence resonance energy transfer to monitor G protein activation and CRISPR/Cas9 gene-edited THP-1 cells lacking AVPR1A or α_1B_-AR, we show that CRs that share the propensity to heteromerize with α_1B/D_-ARs and AVPR1A exist and function within interdependent hetero-oligomeric complexes through which the efficacy of CRs to mediate chemotaxis is controlled. Our findings suggest that hetero-oligomers composed of CRs, α_1B/D_-ARs, and AVPR1A may enable stress hormones to regulate immune cell trafficking.

## Abstract (precis)

Many chemokine receptors in human monocytes function within hetero-oligomeric complexes with α_1_-adrenoceptors and arginine vasopressin receptor 1A through which the chemokine receptor heteromerization partners are regulated.

## Introduction

The super-family of seven-transmembrane (7TM) receptors, of which most are G protein–coupled receptors (GPCRs), are critical mediators of numerous physiological and pathological processes in the human body. Accordingly, GPCRs are the largest protein family targeted by Federal Drug Administration–approved drugs (1, 2, 3).

There is growing evidence that many GPCRs may form heterodimers and higher order hetero-oligomeric complexes, which exhibit pharmacological behavior distinct from their individual protomers (4, 5, 6, 7, 8, 9, 10). The extent to which GPCRs are able to form heteromers and the potential physiological relevance of such receptor complexes, however, are not well understood.

Previously, we provided evidence that numerous chemokine receptors (CRs) form heteromeric complexes with α_1_-adrenergic receptors (α_1_-ARs) in recombinant systems, in rodent and human vascular smooth muscle cells, in the human monocytic cell line THP-1, and in freshly isolated human monocytes through which the receptor partners modulate their function (9, 11, 12, 13, 14, 15, 16, 17, 18). Moreover, we reported that arginine vasopressin receptor 1A (AVPR1A) also heteromerizes with chemokine (C-X-C motif) receptor 4 (CXCR4), atypical chemokine receptor 3 (ACKR3), and α_1_-ARs in recombinant systems and in human vascular smooth muscle cells (9, 13, 19). It is unknown, however, whether heteromers composed of CRs and AVPR1A are expressed in leukocytes, whether other members of the human CR family also form heteromers with AVPR1A, and whether AVPR1A influences CR function.

The aims of the present study were to assess whether AVPR1A may form heteromers with other members of the CR family and to obtain initial insights into the potential functional roles of such heteromers. Thus, we employed bioluminescence resonance energy transfer (BRET) to characterize the heteromerization interactome between AVPR1A and all 23 human CRs and to evaluate CR-mediated G protein activation in a recombinant system, and used the human monocytic cell line THP-1 and freshly isolated human monocytes as cell models to evaluate the possible roles of such endogenously expressed heteromers in the regulation of CR function.

## Results

### AVPR1A heteromerizes with numerous CRs in a HEK293T expression system

**Figure 1.**
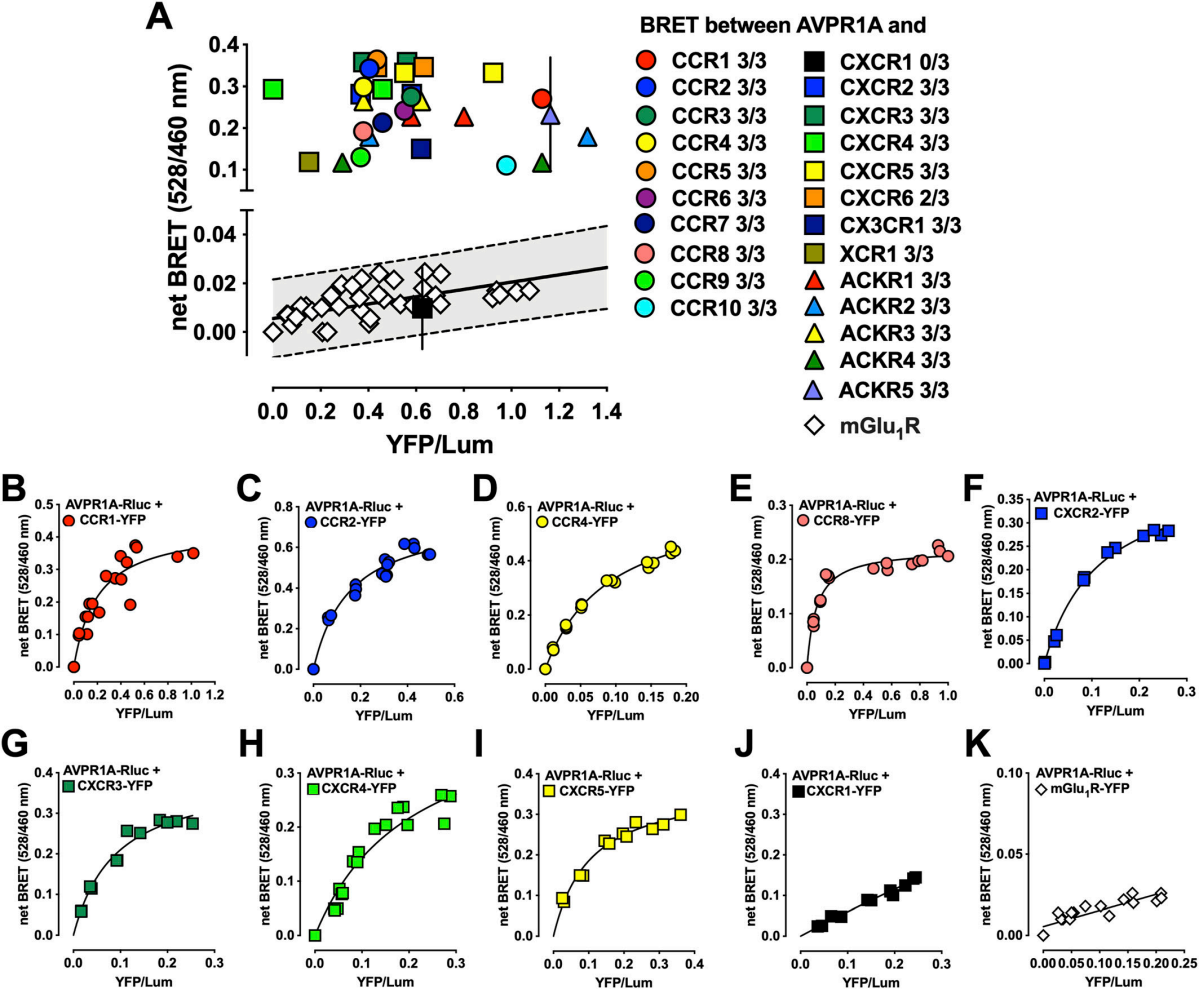
Bioluminescence resonance energy transfer (BRET) for the identification of chemokine receptor (CR) heteromerization partners of AVPR1A. YFP fluorescence and luminescence were read as described in the Materials and Methods section. Net BRET (528/460 nm) was plotted against YFP fluorescence/luminescence (YFP/Lum). **(A)** HEK293T cells were transfected with AVPR1A-Rluc plus each CR-YFP in triplicate. Net BRET signals are the mean ± SD. Cells transfected with AVPR1A-Rluc and mGlu_1_R-YFP at various energy acceptor:donor ratios served as nonspecific controls; nonspecific BRET signals were analyzed by linear regression analysis. The black line shows the regression line, and the dashed lines indicate 99% prediction bands. The gray area indicates the expected distribution of nonspecific BRET signals. BRET signals above the 99% prediction band for nonspecific interactions were considered positive signals for interactions between CRs (colored symbols) and AVPR1A. The graph represents one of three screening experiments. The number of positive BRET signals in three independent screening experiments (0–3/3) is shown for each CR. **(B, C, D, E, F, G, H, I, J, K)** HEK293T cells were transfected with a fixed amount of AVPR1A-Rluc and with increasing amounts of CR-YFP in duplicate. Figures show saturation BRET signals representative of n = 3 independent experiments per receptor pair. Saturation BRET between AVPR1A and CCR1 (B), CCR2 (C), CCR4 (D), CCR8 (E), CXCR2 (F), CXCR3 (G), CXCR4 (H), CXCR5 (I), CXCR1 (J), and mGlu_1_R-YFP (K).

To evaluate the interactome between AVPR1A and the family of CRs, we screened for receptor–receptor interactions using BRET. BRET screening experiments were performed as previously described for the assessment of interactions between CRs and α_1_-ARs (14). HEK293T cells were transfected with AVPR1A C-terminally ligated to the energy donor *Renilla luciferase* (AVPR1A-Rluc) plus one of the 23 CRs C-terminally ligated to the energy acceptor enhanced yellow fluorescent protein (CR-YFP). As a control, cells were transfected with AVPR1A-Rluc plus metabotropic glutamate receptor 1 (mGlu_1_R)-YFP at various energy donor:acceptor ratios (14, 16). We considered BRET signals for interactions between AVPR1A and CRs above the 99% prediction band for signals between AVPR1A and mGlu_1_R as positive heteromerization signals. Fig 1A shows a representative BRET screening experiment and provides the number of positive signals for each CR-AVPR1A combination in three independent screening experiments. We observed positive BRET signals in all three screening experiments for 21 of the 23 members of the CR family and in two of the three screening experiments for CXCR6. BRET interaction signals for CXCR1 were negative in all three screening experiments.

To confirm these findings, we selected several CRs that showed positive interaction signals in the screening experiments and CXCR1, and performed saturation BRET experiments (14). As shown in Fig 1B–K, we detected hyperbolic progressions of the BRET signals with increasing energy acceptor:donor ratios for interactions between AVPR1A-Rluc and CCR1-YFP (Fig 1B), CCR2-YFP (Fig 1C), CCR4-YFP (Fig 1D), CCR8-YFP (Fig 1E), CXCR2-YFP (Fig 1F), CXCR3-YFP (Fig 1G), CXCR4-YFP (Fig 1H), and CXCR5-YFP (Fig 1I). In contrast, BRET signals between AVPR1A and CXCR1 (Fig 1J) and between AVPR1A and mGlu_1_R (Fig 1K) increased linearly with increasing energy acceptor: donor ratios, which is consistent with nonspecific bystander BRET signals.

### Detection of AVPR1A:CR heteromers in THP-1 cells and human monocytes

**Figure 2.**
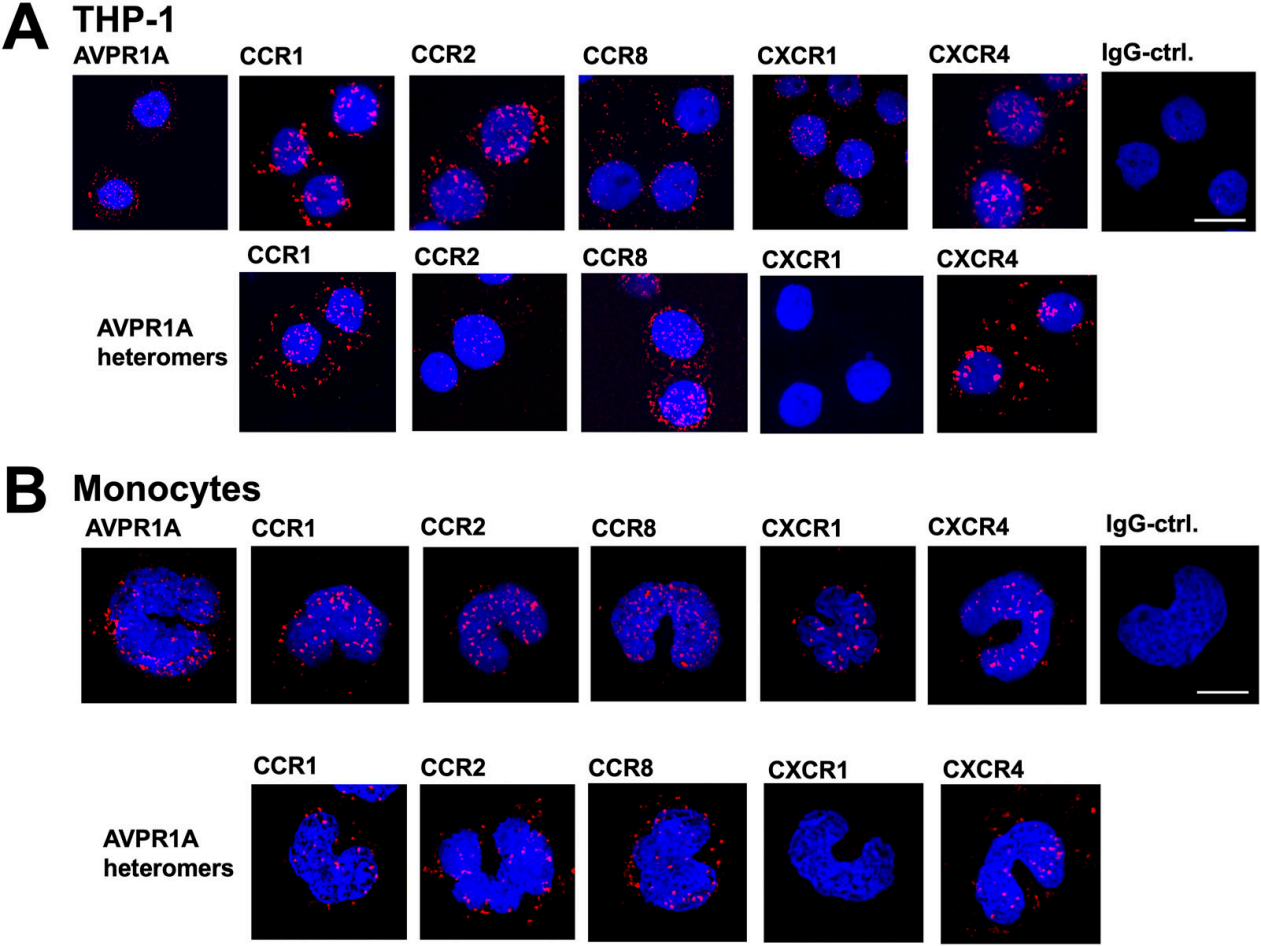
CR:AVPR1A heteromers are detectable in THP-1 cells and human monocytes. **(A, B)** Representative proximity ligation assay images for the detection of individual receptors ((A, B), top rows) and receptor–receptor proximity ((A, B), bottom rows) in THP-1 cells (A) and human monocytes (B). Images show merged DAPI (nuclear counterstain) and proximity ligation assay signals (red, λ_excitation/emission_ 598/634 nm) acquired from z-stack images (n = 10; thickness = 0.5 μm, bottom to top) and are representative of n = 3 independent experiments. As controls, cells were incubated with IgG (A/B, top) or a combination of IgG and anti-AVPR1A (not shown). **(A, B)** Scale bars, (A) 10 μm and (B) 5 μm.

To determine whether our findings from a recombinant expression system apply to endogenous systems of expression, we performed proximity ligation assays (PLAs) to visualize individual receptors and proximity between two receptor partners in the human monocytic leukemia cell line THP-1 (Fig 2A) and in freshly isolated human monocytes (Fig 2B). AVPR1A and the selected CRs CCR1, CCR2, CCR8, CXCR1, and CXCR4 could be visualized individually by the PLA in THP-1 cells (Fig 2A, top row) and in monocytes (Fig 2B, top row). When the PLA was performed to visualize receptor–receptor proximity, we observed positive signals for proximity between AVPR1A and CCR1, CCR2, CCR8, and CXCR4 in THP-1 cells (Fig 2A, bottom row) and in monocytes (Fig 2B, bottom row). In contrast, we did not observe PLA signals for proximity between AVPR1A and CXCR1 (Fig 2A and B, bottom rows).

### AVPR1A ligands modulate chemotaxis mediated by CR heteromerization partners

**Figure 3.**
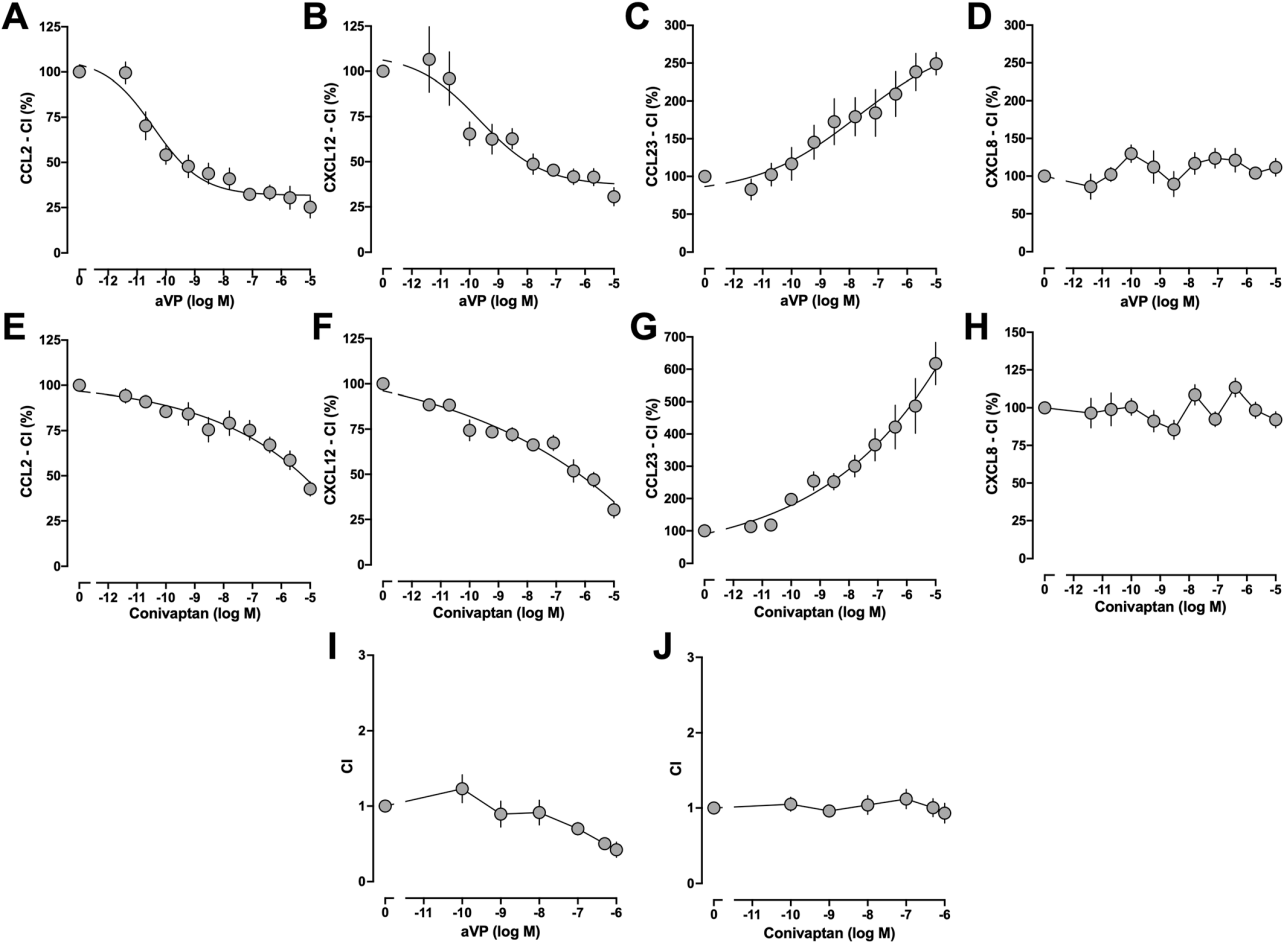
AVPR1A ligands modulate chemotaxis mediated by CR heteromerization partners of AVPR1A in THP-1 cells. **(A, B, C, D, E, F, G, H)** THP-1 cells were exposed to various concentrations of arginine vasopressin (aVP) (A, B, C, D) or conivaptan (E, F, G, H), and chemotaxis toward CCL2 (10 nmol/liter, (A, E)), CXCL12 (100 nmol/liter, (B, F)), CCL23 (0.1 nmol/liter, (C, G)), or CXCL8 (10 nmol/liter, (D, H)) was tested. CI (%), chemotactic index in the percentage of cells not exposed to AVPR1A ligands. Data are the mean ± SE from n = 3–4 independent experiments. **(I, J)** Migration of THP-1 cells toward various concentrations of aVP (I) and conivaptan (J). CI, chemotactic index. Data are the mean ± SE from n = 3 independent experiments.

To test whether AVPR1A ligands modulate the function of CR heteromerization partners in transwell migration assays (Fig 3), cells were exposed to various concentrations of arginine vasopressin (aVP) or the pan-AVPR antagonist conivaptan, and chemotaxis toward the chemokines was tested. Exposure of THP-1 cells to aVP dose-dependently inhibited chemotaxis induced by CCL2 (Fig 3A), the principal endogenous agonist of CCR2, and chemotaxis induced by the CXCR4 agonist CXCL12 (Fig 3B), respectively, with high potency (IC_50_: CCL2—42 ± 21 pM; CXCL12—250 ± 262 pM) and more than 60% efficacy. In contrast, exposure of THP-1 cells to aVP dose-dependently enhanced chemotaxis induced by the CCR1 agonist CCL23 to 278% ± 183% of cells not exposed to aVP (Fig 3C). The EC_50_ of aVP to enhance CCR1-mediated chemotaxis was 17 ± 7 nM. aVP, however, did not affect chemotaxis toward CXCL8, an endogenous agonist of CXCR1 and CXCR2 (Fig 3D).

Exposure of cells to conivaptan also inhibited CCL2 (Fig 3E)- and CXCL12 (Fig 3F)-induced chemotaxis, enhanced CCL23 (Fig 3G)-induced chemotaxis, and did not affect CXCL8-induced chemotaxis (Fig 3H). The IC_50_ for the inhibitory effects of conivaptan on CCL2- and CXCL12-induced chemotaxis, as well as the EC_50_ for its enhancing effects on CCL23-induced chemotaxis, could not be determined because the dose–response curves did not reach the bottom or top plateaus, respectively, in these experiments. Because aVP (Fig 3I) and conivaptan (Fig 3J) did not exhibit chemoattractant or chemorepellent activity in THP-1 cells, the observed effects of the AVPR1A ligands cannot be attributed to reversed chemotactic gradients in our experiments.

**Figure 4.**
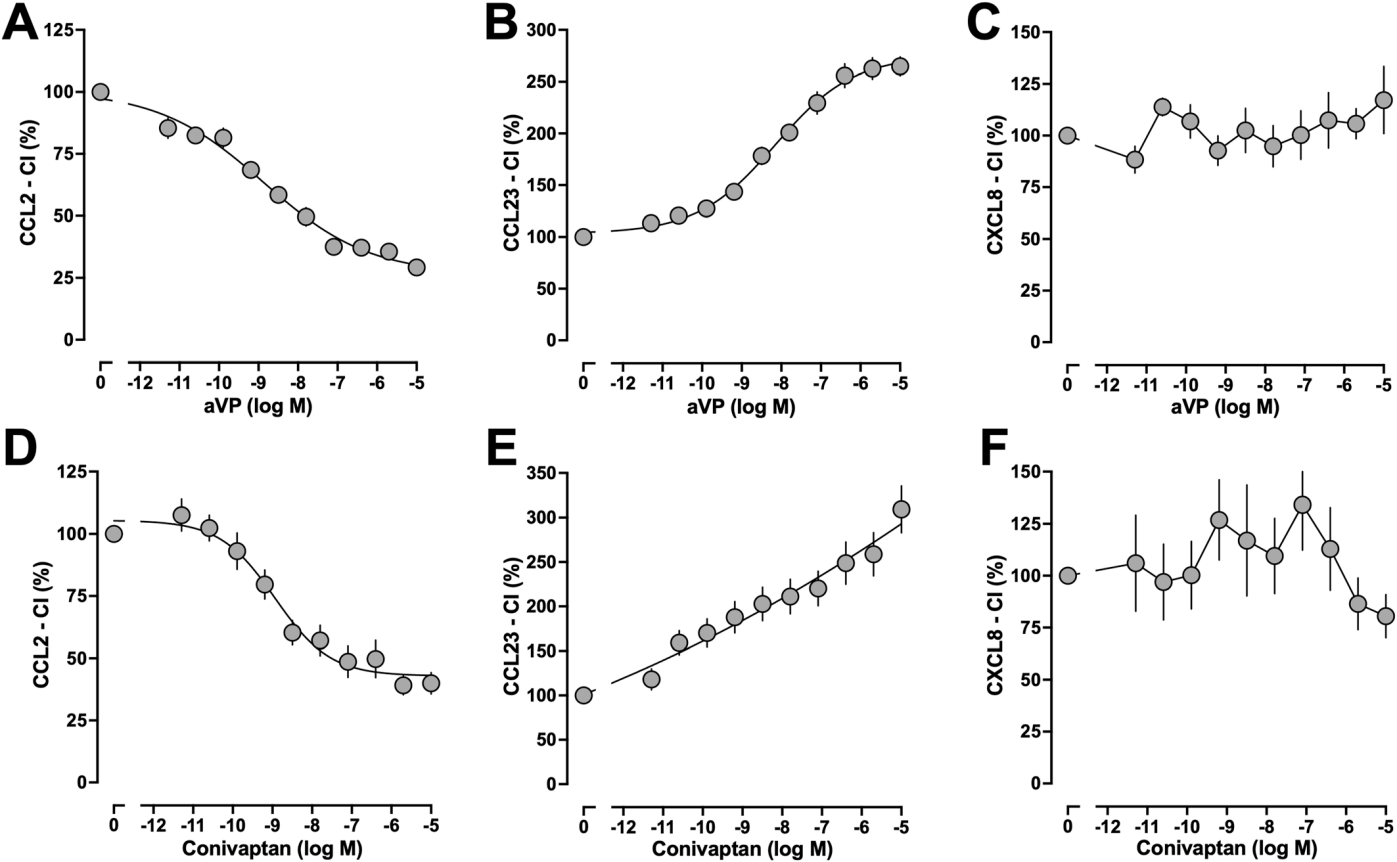
AVPR1A ligands modulate chemotaxis mediated by CR heteromerization partners of AVPR1A in human monocytes. **(A, B, C, D, E, F)** Human monocytes were exposed to various concentrations of aVP (A, B, C) or conivaptan (D, E, F), and chemotaxis toward CCL2 (10 nmol/liter, (A, D)), CCL23 (0.1 nmol/liter, (B, E)), and CXCL8 (10 nmol/liter, (C, F)) was tested. CI (%), chemotactic index in the percentage of cells not exposed to AVPR1A ligands. Data are the mean ± SE from n = 3–4 independent experiments.

As observed in THP-1 cells, we detected comparable efficacies and potencies of aVP (Fig 4A–C) and conivaptan (Fig 4D–F) to modulate chemotaxis in freshly isolated human monocytes. While aVP dose-dependently (IC_50_: 1 ± 0.7 nM) inhibited CCL2-induced chemotaxis by 74% ± 4% (Fig 4A), aVP dose-dependently (EC_50_: 7 ± 3 nM) enhanced CCL23-induced chemotaxis by 275% ± 9% (Fig 4B) and did not affect CXCL8-induced chemotaxis (Fig 4C). Similarly, conivaptan dose-dependently (IC_50_: 1 ± 0.6 nM) inhibited CCL2-induced chemotaxis by 58% ± 4% (Fig 4D), dose-dependently enhanced CCL23-induced chemotaxis by 309% ± 26% (Fig 4E), and did not significantly affect CXCL8-induced chemotaxis (Fig 4F). As for the effects of conivaptan on CCL23-induced chemotaxis in THP-1 cells, the EC_50_ of its enhancing effects on CCL23-induced chemotaxis in monocytes could not be determined.

**Figure 5.**
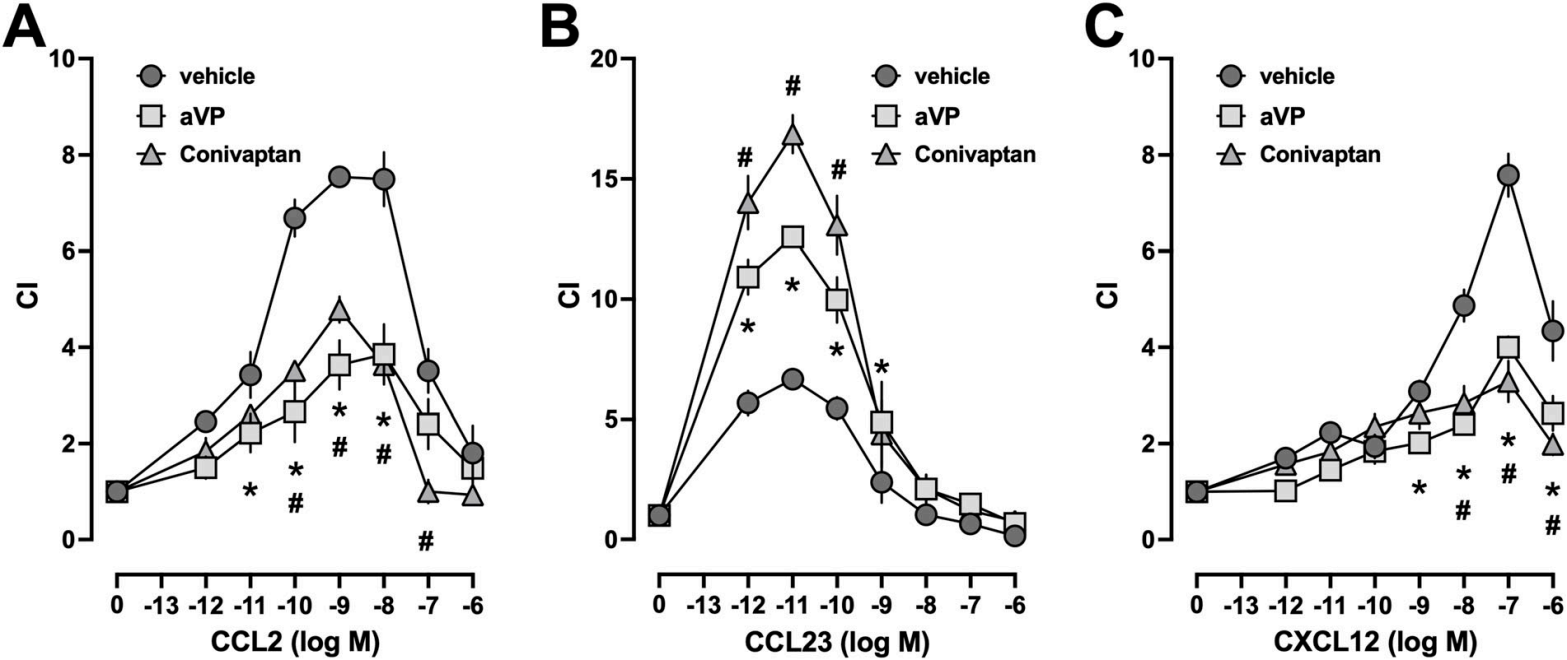
AVPR1A ligands modulate the efficacy of CR heteromerization partners of AVPR1A to mediate chemotaxis. **(A, B, C)** Chemotactic dose–responses for CCL2 ((A), n = 3), CCL23 ((B), n = 6), and CXCL12 ((C), n = 3) in THP-1 cells exposed to 10 μmol/liter arginine vasopressin (aVP), 10 μmol/liter conivaptan, or vehicle. Data are the mean ± SE. *P < 0.05 for aVP versus vehicle; ^#^*P* < 0.05 for conivaptan versus vehicle (two-way ANOVA with Dunnett’s multiple comparisons test).

To further assess whether the AVPR1A ligands affect the potency of the chemokines to induce chemotaxis, we determined the dose–response profiles of CCL2 (Fig 5A), CCL23 (Fig 5B), and CXCL12 (Fig 5C) in THP-1 cells that were exposed to vehicle or 10 μM of aVP or conivaptan. Although aVP and conivaptan inhibited cell migration toward CCL2 and CXCL12, both AVPR1A ligands enhanced migration toward CCL23 without affecting the bell-shaped dose–response profiles of the chemokines.

### AVPR1A ligands interfere with heteromerization between AVPR1A and its CR partners

**Figure 6.**
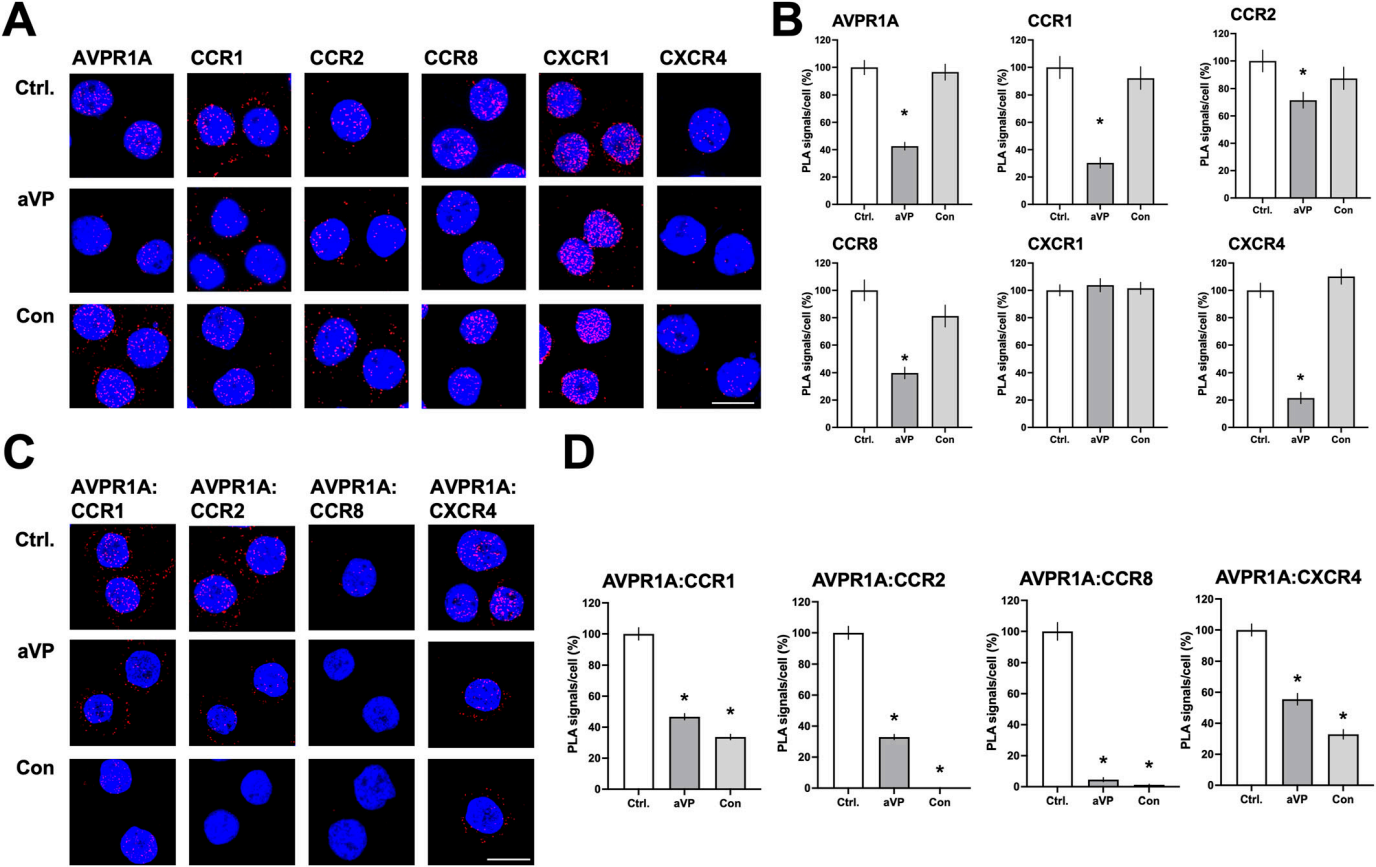
AVPR1A ligands interfere with heteromerization between AVPR1A and CR partners. **(A, C)** THP-1 cells were incubated with vehicle (ctrl., top), 10 μmol/liter arginine vasopressin (aVP, center), or 10 μmol/liter conivaptan (con, bottom) for 30 min at 37°C, and the cell surface expression of individual receptors (A) and receptor–receptor proximity (C) was visualized by the proximity ligation assay (PLA). Images show merged DAPI (nuclear counterstain) and PLA signals (red, λ_excitation/emission_ 598/634 nm) acquired from z-stack images (n = 10; thickness 0.5 μm, bottom to top) and are representative of n = 3 independent experiments. Scale bar, 10 μm. **(B)** Quantification of PLA signals for AVPR1A, CCR1, CCR2, CCR8, CXCR1, and CXCR4 from n = 3 experiments. Data are the mean ± SE. *P < 0.05 versus ctrl. **(D)** Quantification of PLA signals for receptor–receptor proximity between AVPR1A and CCR1, CCR2, CCR8, or CXCR4 from n = 3 experiments. Data are the mean ± SE. *P < 0.05 versus ctrl.

Next, we used the PLA to test whether aVP and conivaptan affect the formation of AVPR1A:CR heteromers in THP-1 cells. Fig 6A shows representative PLA images for the detection of individual receptors in THP-1 cells exposed to vehicle (ctrl.) or 10 μM of aVP or conivaptan, and Fig 6B shows the quantification of PLA signals from three independent experiments. As compared to vehicle-treated cells, conivaptan did not affect PLA signals for any of the receptors. In contrast, PLA signals for AVPR1A and the heteromerization partners CCR1, CCR2, CCR8, and CXCR4 were significantly reduced in aVP-treated cells, whereas PLA signals for CXCR1 were not affected.

Representative PLA images for the detection of receptor–receptor proximity in THP-1 cells exposed to vehicle (ctrl.) or 10 μM of aVP or conivaptan are shown in Fig 6C, and Fig 6D shows the quantification of PLA signals from three independent experiments. aVP and conivaptan significantly reduced PLA signals for proximity between AVPR1A and CCR1, CCR2, CCR8, or CXCR4, when compared to vehicle-treated cells.

### Reduction of AVPR1A expression and lack of AVPR1A modulate chemotaxis mediated by CR heteromerization partners

**Figure 7.**
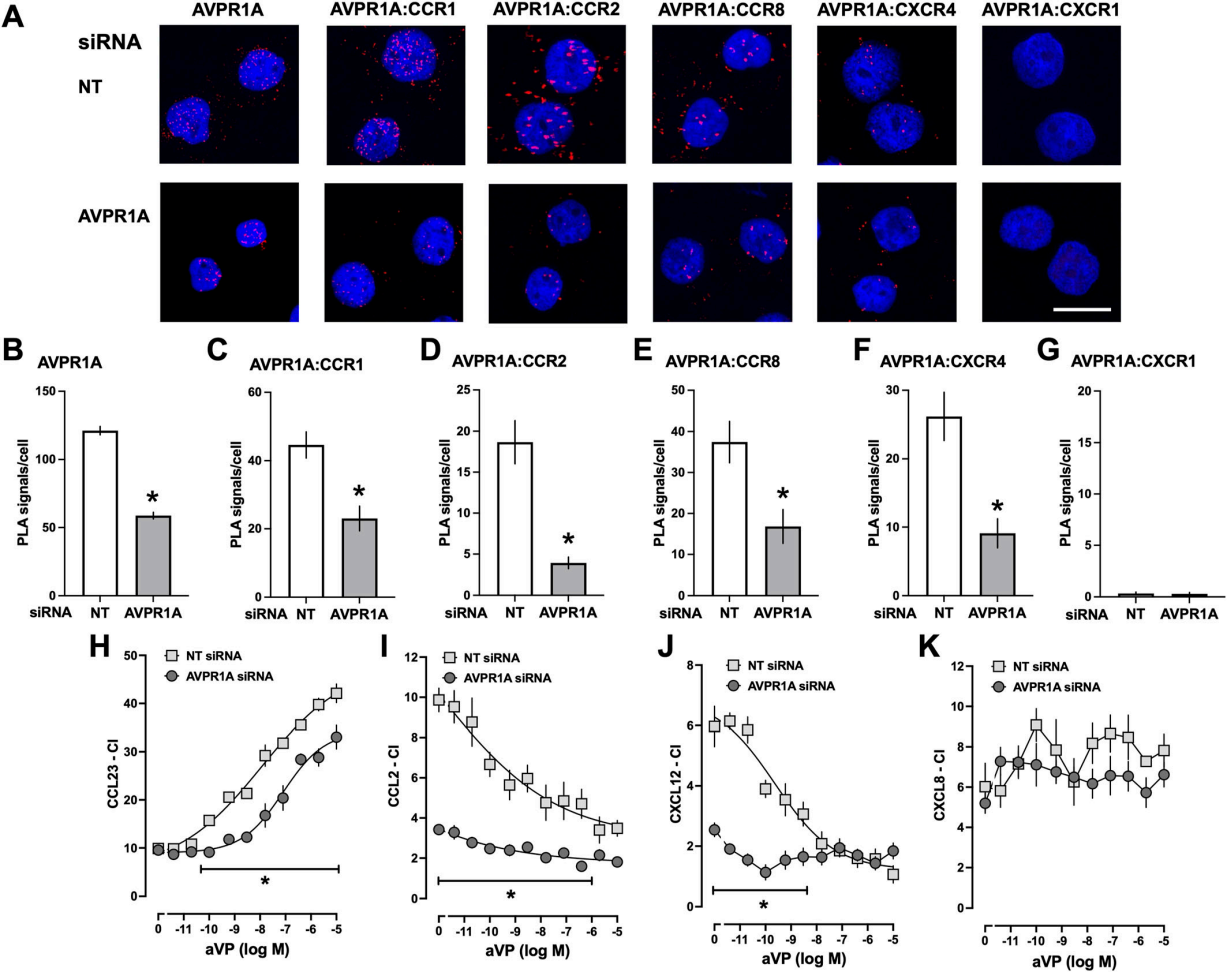
siRNA knockdown of AVPR1A modulates chemotaxis mediated by CR heteromerization partners of AVPR1A. **(A, B, C, D, E, F, G)** THP-1 cells were incubated with NT or AVPR1A siRNA. **(A)** Representative images for the detection of AVPR1A and proximity between AVPR1A and CCR1, CCR2, CCR8, CXCR4, and CXCR1 by the proximity ligation assay (PLA). Images show merged DAPI/PLA signals and are representative of n = 3 independent experiments. Scale bar, 10 μm. **(B, C, D, E, F, G)** Quantification of the number of PLA signals per cell for AVPR1A and receptor–receptor proximities in THP-1 cells after incubation with NT siRNA (ctrl., white bars) or AVPR1A siRNA (gray bars) (n = 3). Data are the mean ± SE. *P < 0.05 versus cells treated with NT siRNA. **(H, I, J, K)** THP-1 cells were incubated with NT or AVPR1A siRNA as in (A, B, C, D, E, F, G). Cells were then exposed to various concentrations of aVP, and chemotaxis toward CCL23 (0.1 nmol/liter, (H)), CCL2 (10 nmol/liter, (I)), CXCL12 (100 nmol/liter, (J)), and CXCL8 (10 nmol/liter, (K)) was tested. CI, chemotactic index (mean ± SE, n = 3/condition). *P < 0.05 versus cells incubated with NT siRNA (two-way ANOVA with Dunnett’s multiple comparisons test).

Because our findings on the effects of AVPR1A ligands may suggest that interference with AVPR1A:CR heteromerization regulates the function of the CR partners, we tested how depletion of AVPR1A from the cell surface via siRNA gene silencing affects chemotaxis mediated by the corresponding CRs. Representative PLA images for the detection of AVPR1A and of proximity between AVPR1A and selected CRs in THP-1 cells after incubation with nontargeting (NT) or AVPR1A siRNA are shown in Fig 7A, and Fig 7B–G show the quantifications of PLA signals from three independent experiments. Incubation of cells with AVPR1A siRNA reduced PLA signals for AVPR1A by 48.5% ± 2.5%, as compared to cells incubated with NT siRNA (Fig 7B). It should be noted that we did not analyze the expression of individual CRs in these experiments because we showed previously that AVPR1A siRNA does not affect expression levels of CXCR4, ACKR3, or any α_1_-AR subtype (13). As expected, in cells exposed to AVPR1A siRNA, PLA signals for proximity between AVPR1A and its heteromerization partners CCR1 (Fig 7C), CCR2 (Fig 7D), CCR8 (Fig 7E), and CXCR4 (Fig 7F) were reduced to a degree comparable to the reduction of PLA signals for AVPR1A, when compared to cells treated with NT siRNA. PLA signals for proximity between AVPR1A and CXCR1 were not detectable in cells incubated with NT or AVPR1A siRNA (Fig 7G).

The chemotactic behavior of THP-1 cells after incubation with NT and AVPR1A siRNA in response to the various chemokines and the effects of aVP on those responses are shown in Fig 7H–K. As compared to cells exposed to NT siRNA, chemotaxis toward CCL23 was not affected by AVPR1A siRNA (Fig 7H). However, the potency and efficacy of aVP to enhance CCL23-induced chemotaxis were significantly reduced after partial siRNA knockdown of AVPR1A (EC_50_: NT siRNA—11 ± 6 nM; AVPR1A-siRNA—80 ± 44 nM, *P* < 0.05; top plateau: NT siRNA—46 ± 3 CI; AVPR1A-siRNA—34 ± 2.5 CI, *P* < 0.05; Fig 7H). In contrast to CCL23, chemotaxis toward CCL2 (Fig 7I) and CXCL12 (Fig 7J) was significantly reduced after incubation of cells with AVPR1A siRNA, when compared to cells incubated with NT siRNA (CI CCL2: NT siRNA 9.7 ± 0.6, AVPR1A siRNA 3.4 ± 0.2, *P* < 0.05; CI CXCL12: NT siRNA 6 ± 0.7, AVPR1A siRNA 2.5 ± 0.2, *P* < 0.05). As observed for CCL23, however, the efficacy of aVP to inhibit CCL2- and CXCL12-mediated chemotaxis was reduced in cells after incubation with AVPR1A siRNA (% inhibition at 10 μM aVP: CCL2—NT siRNA 65% ± 4%, AVPR1A siRNA 47% ± 5%, *P* < 0.05; CXCL12—NT siRNA 82% ± 5%, AVPR1A siRNA 30% ± 8%, *P* < 0.05). The potency of aVP to inhibit CCL2- and CXCL12-induced chemotaxis in cells after incubation with AVPR1A siRNA could not be determined with confidence because of the low chemotactic activity of the chemokines. As anticipated, chemotaxis toward CXCL8 was indistinguishable between THP-1 cells incubated with NT and AVPR1A siRNA and not affected by aVP (Fig 7K).

**Figure 8.**
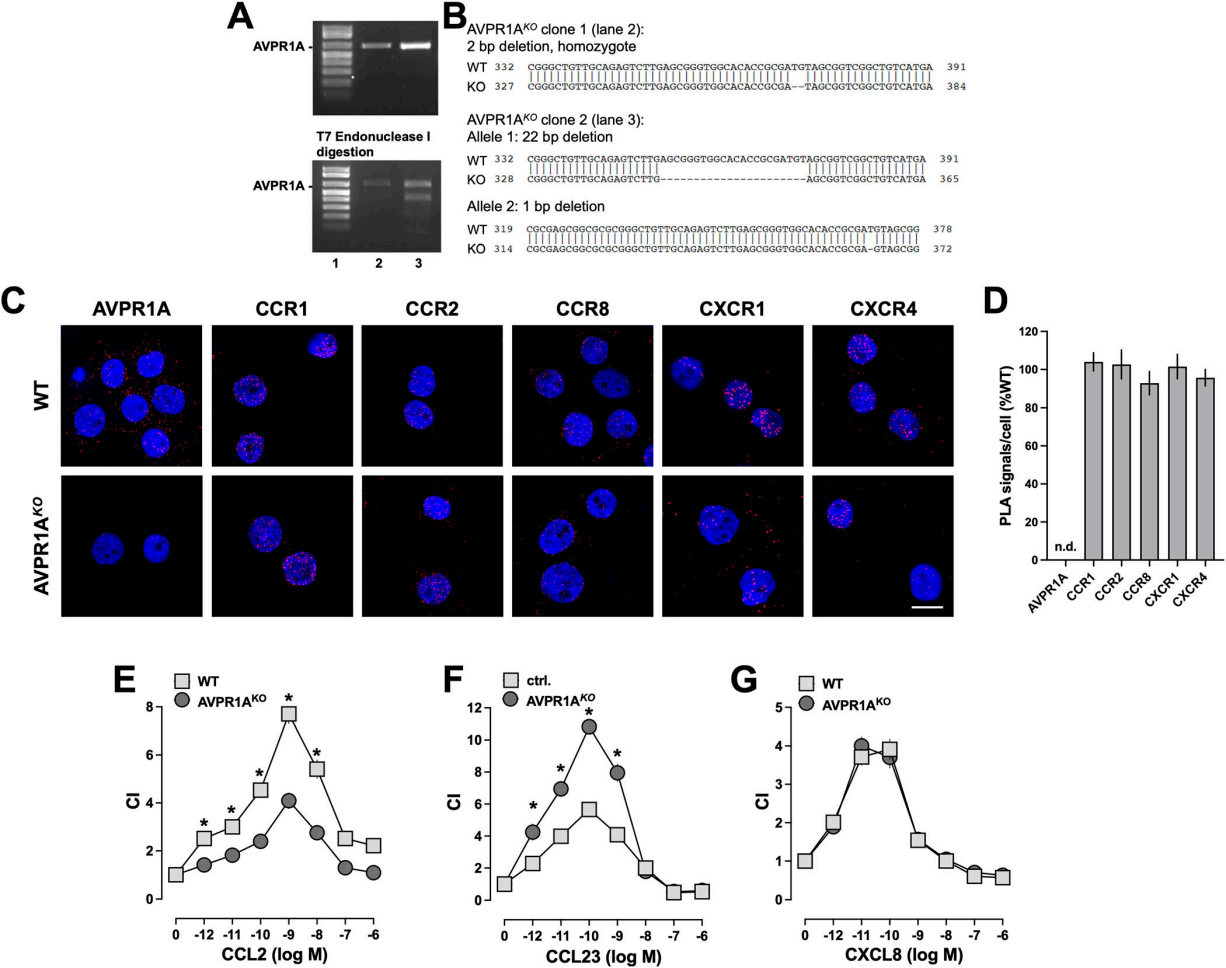
CRISPR/Cas9 knockout of AVPR1A modulates chemotaxis mediated by CR heteromerization partners of AVPR1A. **(A, B)** CRISPR/Cas9 gene editing to generate THP-1 cell lines that lack AVPR1A, with the designation AVPR1A^*KO*^. **(A)** T7 surveyor assay. Images from agarose gel electrophoresis for the detection of PCR-amplified AVPR1A genomic DNA before (top) and after (bottom) T7EI digestion from puromycin-selected THP-1 cell clones that were transduced with the lentivirus encoding sgRNA targeting AVPR1A and Cas9 (lanes 2 and 3). Lane 1: DNA ladder. **(B)** Scheme depicting the modified genomic region of AVPR1A in AVPR1A^*KO*^ clones from lanes 2 and 3. **(C)** Detection of individual receptors in a WT THP-1 clone (WT, top) and AVPR1A^KO^ (bottom) by the proximity ligation assay (PLA). Images show merged DAPI/PLA signals and are representative of n = 3 independent experiments. Scale bars, 10 μm. **(D)** Quantification of PLA signals per cell for the detection of individual receptors in AVPR1A^*KO*^ cells. Data (mean ± SE) are expressed as the percentage of a WT THP-1 cell clone (% WT). *P < 0.05 versus ctrl. **(E, F, G)** Chemotaxis of AVPR1A^*KO*^ and WT THP-1 clones toward various concentrations of CCL2 (E), CCL23 (F), and CXCL8 (G). CI, chemotactic index (mean ± SE, n = 3–4 independent experiments). *P < 0.05 for AVPR1A^*KO*^ versus WT THP-1 clones (two-way ANOVA with Dunnett’s multiple comparisons test).

To confirm these findings and preclude limitations of partial AVPR1A knockdown, we generated THP-1 cell lines that lack AVPR1A using CRISPR/Cas9 gene editing. Fig 8A shows the PCR-amplified AVPR1A genomic DNA before and after T7 endonuclease I (T7EI) digestion from two puromycin-selected THP-1 cell clones that were transduced with the lentivirus encoding single guide RNA (sgRNA) targeting AVPR1A and Cas9. Fig 8B shows the results from the sequencing of the plasmids after subcloning of PCR-amplified DNA from these cell clones to the thymine adenine (TA) cloning vector. We generated and expanded two clones that lack AVPR1A: AVPR1A^*KO*^ clone 1 showed a homozygous 2 base pair (bp) deletion, which does not result in visible mismatch products after T7EI digestion (Fig 8A, lane 2). AVPR1A^*KO*^ clone 2 (Fig 8A, lane 3) showed a 22-bp deletion in allele 1 and a 1-bp deletion in allele 2. To exclude clonal-specific artifacts, all subsequent experiments with AVPR1A^*KO*^ cells were performed with both clones and revealed identical findings. Fig 8C shows representative PLA images for the detection of AVPR1A, CCR1, CCR2, CCR8, CXCR1, and CXCR4, and Fig 8D shows the quantification of PLA signals from three independent experiments. As anticipated, AVPR1A was not detectable in AVPR1A^*KO*^ cells and the expression of the CRs was not affected, when compared to a THP-1 cell clone that showed no change in the AVPR1A sequence (=ctrl.). The chemotactic behavior of THP-1 control cells and AVPR1A^*KO*^ cells in response to CCL2, CCL23, and CXCL8 is shown in Fig 8E–G, respectively. We observed that lack of AVPR1A significantly inhibits chemotaxis toward CCL2 (Fig 8E) and enhances chemotaxis toward CCL23 (Fig 8F). Lack of AVPR1A did not affect chemotaxis toward CXCL8 (Fig 8G). The absence of AVPR1A did not affect the potency of the chemokines to induce chemotaxis.

### CR:AVPR1A and CR:α_1B/D_-AR heteromers form and function interdependently

To evaluate whether CRs may exist and function within hetero-oligomeric complexes composed of AVPR1A and α_1B/D_-ARs, we used the PLA to analyze CR:α_1B/D_-AR heteromerization in AVPR1A^*KO*^ cells and CR:AVPR1A heteromerization in ADRA1B^*KO*^ cells. ADRA1B^*KO*^ cells lack α_1B_-AR, and show more than 80% reduction of α_1D_-AR expression and unaffected expression of individual CRs, when compared to control THP-1 cells (14).

**Figure 9.**
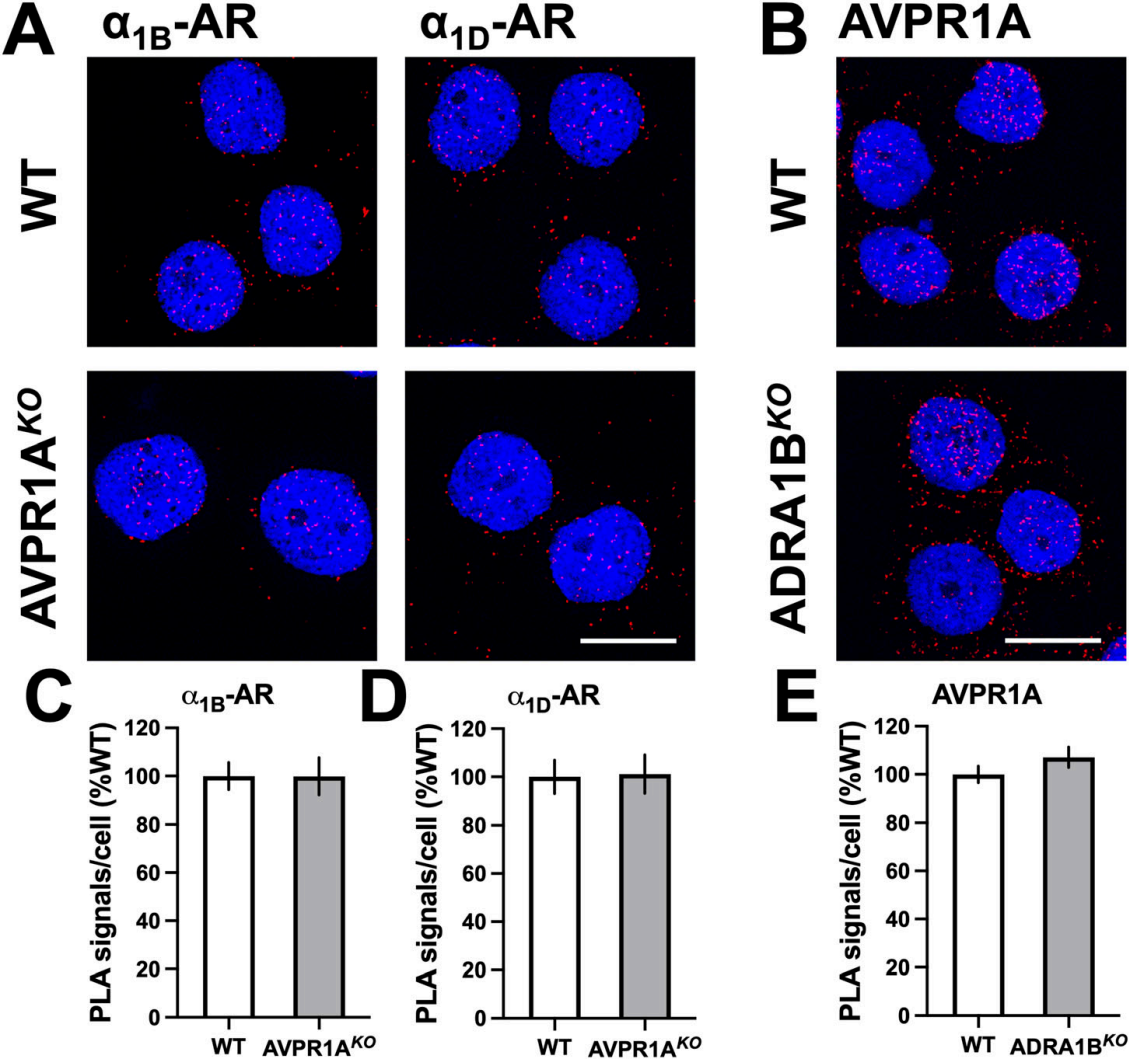
CRISPR/Cas9 knockout of AVPR1A or ADRA1B does not affect -α_1B/D_-AR or AVPR1A expression, respectively. Expression of individual receptors by the proximity ligation assay (PLA). Images show merged DAPI/PLA signals and are representative of n = 3 independent experiments. Scale bars, 10 μm. **(A)** PLA images for the detection of α_1B/D_-AR in WT and AVPR1A^*KO*^ cells. **(B)** PLA images for the detection of AVPR1A in WT and AVPR1A^*KO*^ cells. Quantification of PLA signals for α_1B_-AR (C) and α_1D_-AR (D) in WT and AVPR1A^*KO*^ cells and of PLA signals for AVPR1A in WT and ADRA1B^*KO*^-ARs (E) from n = 3 experiments. Data (mean ± SE) are expressed as % of WT, n = 3.

**Figure 10.**
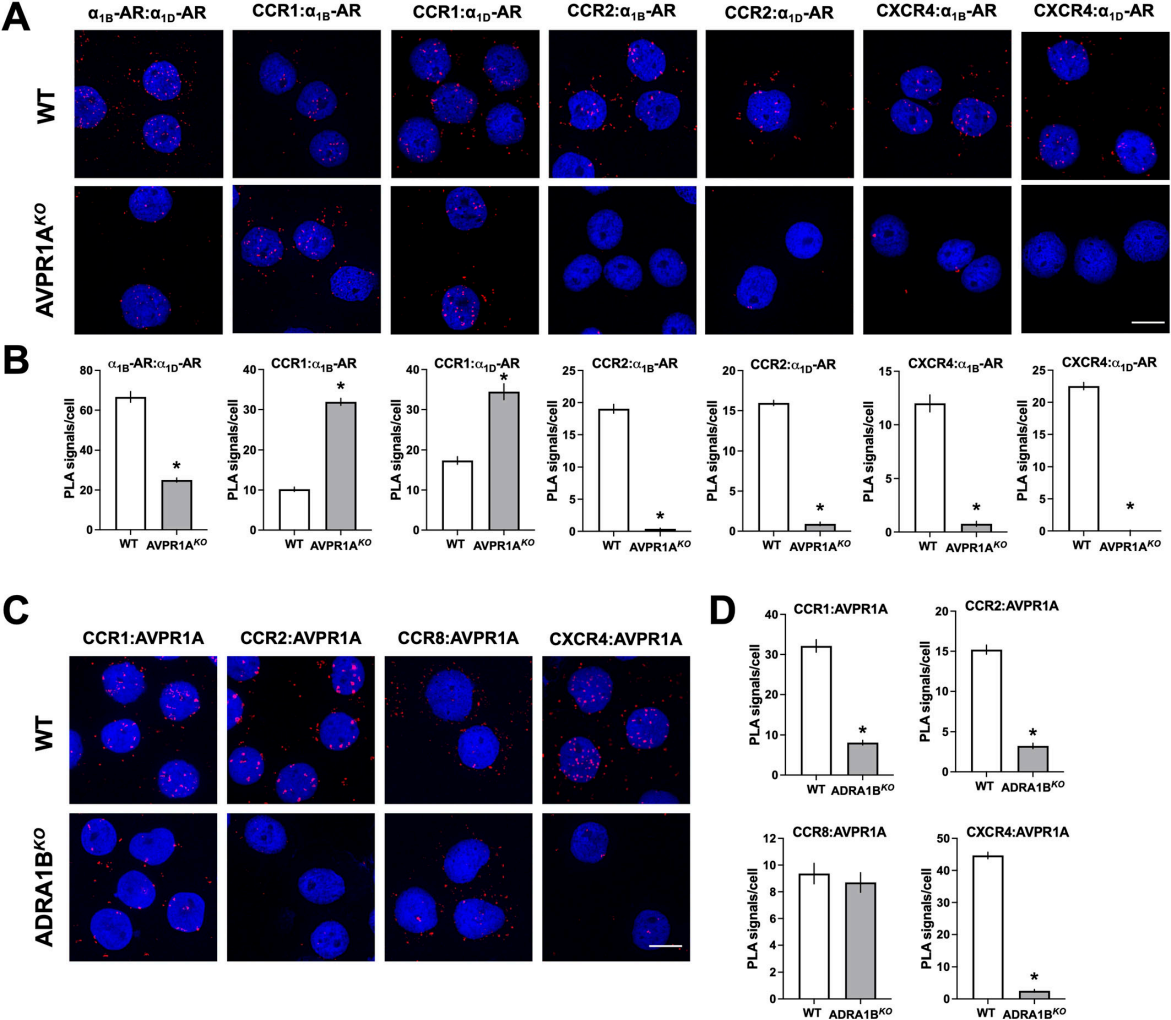
CR:AVPR1A and CR:α_1B_-AR heteromer expression are interdependent. **(A)** Detection of CR:α_1B/D_-AR and α_1B_-AR:α_1D_-AR heteromers in WT THP-1 clones (WT, top) and AVPR1A^*KO*^ clones (bottom) by the proximity ligation assay (PLA). Images show merged DAPI/PLA signals and are representative of n = 3 independent experiments. Scale bars, 10 μm. **(B)** Quantification of PLA signals per cell for the detection of CR:α_1B/D_-AR and α_1B_-AR:α_1D_-AR heteromers in WT THP-1 clones (WT, white bars) and AVPR1A^*KO*^ clones (gray bars). Data are the mean ± SE. *P < 0.05 versus WT THP-1 clones. **(C)** Detection of CR:AVPR1A heteromers in WT THP-1 clones (WT, top) and in ADRA1B^*KO*^ clones (bottom) by the PLA. Images show merged DAPI/PLA signals and are representative of n = 3 independent experiments. Scale bars, 10 μm. **(D)** Quantification of PLA signals per cell for the detection of CR:AVPR1A heteromers in WT THP-1 clones (WT, white bars) and ADRA1B^*KO*^ clones (gray bars). Data are the mean ± SE. *P < 0.05 versus WT THP-1 clones.

The PLA for the detection of individual receptors confirmed that the expression of α_1B/D_-ARs was not affected in AVPR1A^*KO*^ cells (Fig 9A, C, and D) and that AVPR1A expression was not affected in ADRA1B^*KO*^ cells (Fig 9B and E). Fig 10A shows representative PLA images for the detection of proximity between α_1B_-AR and α_1D_-AR and between α_1B/D_-ARs and CCR1, CCR2, and CXCR4 in AVPR1A^*KO*^ and WT control cells, and Fig 10B shows the quantification of the PLA signals from three independent experiments. The absence of AVPR1A significantly reduced PLA signals for proximity between α_1B_-AR and α_1D_-AR and between α_1B/D_-ARs and the CR partners CCR2 and CXCR4. The absence of AVPR1A, however, significantly increased PLA signals for proximity between α_1B/D_-ARs and CCR1. When the PLA was used to detect proximity between AVPR1A and CR partners in ADRA1B^*KO*^ cells (Fig 10C and D), we detected significantly reduced signals for proximity between AVPR1A and CCR1, CCR2, and CXCR4. PLA signals for proximity between AVPR1A and CCR8, a CR that does not heteromerize with α_1B/D_-ARs (14), were not affected in ADRA1B^*KO*^ cells.

**Figure 11.**
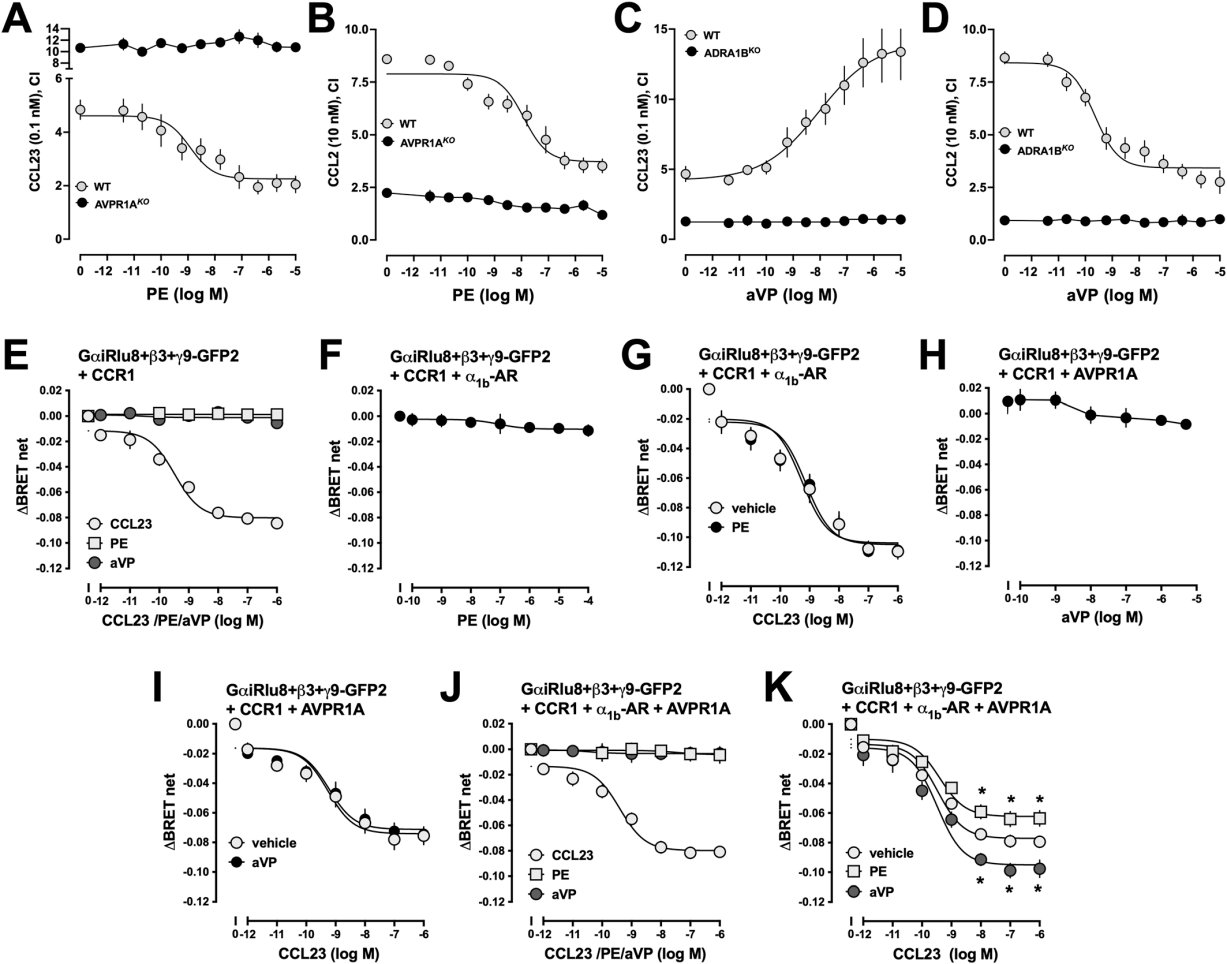
CR:AVPR1A and CR:α_1B/D_-AR heteromers function interdependently. **(A, B)** AVPR1A^*KO*^ and WT THP-1 clones were exposed to various concentrations of phenylephrine, and chemotaxis toward CCL23 (0.1 nmol/liter, (A)) and CCL2 (10 nmol/liter, (B)) was tested. CI, chemotactic index, mean ± SE from n = 3 independent experiments. **(C, D)** ADRA1B^*KO*^ and WT THP-1 clones were exposed to various concentrations of aVP, and chemotaxis toward CCL23 (0.1 nmol/liter (C)) and CCL2 (10 nmol/liter, (D)) was tested. CI, chemotactic index, mean ± SE from n = 3 independent experiments. **(E, F, G, H, I, J, K)** Gαi1 activation assays. Data are the mean ± SE from n = 5 independent experiments. HEK293T cells were transfected with Gαi1-Rlu8, Gβ3, Gγ9-GFP2, and CCR1 together with pcDNA3 (E), α_1b_-AR (F, G), AVPR1A (H/I), or α_1b_-AR plus AVPR1A (J, K). **(E)** Cells were exposed to various concentrations of phenylephrine (PE), aVP, or CCL23. **(F)** Cells were exposed to various concentrations of PE. **(G)** Cells were exposed to various concentrations of CCL23 plus vehicle or 1 μM PE. **(H)** Cells were exposed to various concentrations of aVP. **(I)** Cells were exposed to various concentrations of CCL23 plus vehicle or 0.1 μM aVP. **(J)** Cells were exposed to various concentrations of phenylephrine (PE), aVP, or CCL23. **(K)** Cells were exposed to various concentrations of CCL23 plus vehicle, 1 μM PE, or 0.1 μM aVP. *P < 0.05 versus CCL23 plus vehicle (two-way ANOVA with Dunnett’s multiple comparisons test).

Next, we studied whether the re-arrangements of the CR heteromers in AVPR1A^*KO*^ and ADRA1B^*KO*^ cells modify the effects of α_1B/D_-AR and AVPR1A ligands on CCR1- and CCR2-mediated chemotaxis. We observed that phenylephrine inhibited CCR1 (Fig 11A)- and CCR2 (Fig 11B)-mediated chemotaxis in WT control THP-1 cells (IC_50_: CCR1—1 ± 0.8 nM; CCR2—12 ± 5 nM) by more than 50%, which is consistent with our previous findings (14). In AVPR1A^*KO*^ cells, however, phenylephrine did not affect CCR1- or CCR2-mediated chemotaxis (Fig 11A and B). Similarly, aVP dose-dependently enhanced CCR1-mediated chemotaxis (Fig 11C) and inhibited CCR2-mediated chemotaxis (Fig 11D) in WT control THP-1 cells, but not in ADRA1B^*KO*^ cells (Fig 11C and D).

Furthermore, we employed BRET biosensors to monitor Gαi activation via CCR1 in HEK293T cells expressing CCR1 plus combinations of the receptor partners. In cells expressing CCR1 alone, CCL23 activated Gαi with an EC_50_ of 0.3 ± 0.1 nM, whereas aVP and phenylephrine were ineffective (Fig 11E). In cells co-expressing CCR1 plus α_1b_-AR, phenylephrine did not activate Gαi (Fig 11F) or modulate CCL23-induced Gαi activation (Fig 11G). In cells co-expressing CCR1 plus AVPR1A, aVP did not activate Gαi (Fig 11H) or modulate CCL23-induced Gαi activation (Fig 11I). Although phenylephrine and aVP also did not activate Gαi in cells co-expressing CCR1, α_1b_-AR, and AVPR1A (Fig 11J), phenylephrine significantly reduced the efficacy of CCR1, whereas aVP significantly enhanced the efficacy of CCR1 to activate Gαi upon stimulation with CCL23 (Fig 11K). Phenylephrine and aVP did not affect the EC_50_ of CCL23 to activate Gαi via CCR1.

## Discussion

In the present study, we tested whether recombinant and endogenously expressed AVPR1A is a CR heteromerization partner in HEK293T cells, THP-1 cells, and human monocytes. Our findings from BRET measurements indicate that AVPR1A can form heteromers with all human CRs, except CXCR1, in a recombinant system. The findings that BRET indicated heteromerization between AVPR1A and CXCR2 but not between AVPR1A and CXCR1 were surprising because CXCR1 and CXCR2 share overall 81% sequence similarity, and 79–100% sequence similarity among their transmembrane domains (20, 21). Although structural determinants that facilitate GPCR heteromerization remain to be determined, it might be speculated that AVPR1A heteromerization interfaces in CR heteromerization partners involve other receptor domains, such as extracellular loop 2 or the N- and C-termini, which show lower sequence similarities between CXCR1 and CXCR2 (67%, 31%, and 50%, respectively (20, 21)). To further evaluate possible receptor interaction interfaces, systematic mutagenesis experiments will be required in the future. Nevertheless, the heteromerization interactome that we observed between AVPR1A and CRs is distinct and broader than the previously described interactome between the CR family and each α_1_-AR subtype (14), suggesting that all CR heteromerization partners of α_1_-ARs, except CXCR1, also share the propensity to form heteromers with AVPR1A. In combination with our observations of numerous α_1B/D_-AR:CR and AVPR1A:CR heteromers in THP-1 cells and human monocytes in the present and previous studies (14, 15), our data imply that many CRs constitutively form heteromeric complexes with α_1B/D_-AR and with AVPR1A in monocytes.

Recently, we showed that recombinant hetero-oligomeric complexes composed of CXCR4, ACKR3, α_1_-ARs, and AVPR1A can be formed, which show pharmacological properties distinct from the individual protomers (9, 22). Moreover, we provided evidence that α_1A/B/D_-ARs heteromerize with AVPR1A in a recombinant system and in human vascular smooth muscle cells (9, 13). Thus, CRs that share the heteromerization propensity for α_1B/D_-AR and AVPR1A may exist within heteromers with either α_1B/D_-AR or AVPR1A, or within hetero-oligomeric complexes composed of α_1_-ARs and AVPR1A.

To gain initial insights into the functional relevance of such heteromers in monocytes, we first tested whether AVPR1A ligands affect CR-mediated chemotaxis. Our observations on the effects of aVP and conivaptan on CR-mediated chemotaxis indicate that agonist and antagonist binding to AVPR1A modulate the efficacy of the CR heteromerization partners to induce chemotaxis, but do not affect the potency of the chemokines to induce chemotaxis via their corresponding CRs.

Although our findings suggest that AVPR1A does not heteromerize with recombinant and endogenously expressed CXCR1, BRET indicated that AVPR1A constitutively heteromerizes with CXCR2. CXCL8, however, is an endogenous agonist of CXCR1 and CXCR2, and both receptors are known to be expressed in THP-1 cells and in human monocytes (23, 24). Our observation that the AVPR1A ligands did not affect CXCL8-mediated chemotaxis could be explained by the previous findings that CXCR2 is less frequently expressed than CXCR1 in THP-1 cells and in monocytes, and that selective activation of CXCR2 exhibits only weak chemotactic activity at high chemokine concentrations (24). Moreover, recombinant CXCR1 and CXCR2 have previously been reported to constitutively heterodimerize (25). Thus, the inability of AVPR1A ligands to modulate CXCL8-mediated chemotaxis could also be related to distinct pharmacological properties of putative higher order hetero-oligomeric receptor complexes composed of the CXCR1:CXCR2 heterodimer. Further studies on the possible formation and function of such receptor complexes will be required to address this question. Nevertheless, our finding that both an AVPR1A agonist and antagonist have comparable effects on chemotaxis mediated by CR heteromerization partners of AVPR1A is analogous to the previously described effects of α_1_-AR ligands on CR heteromerization partners of α_1B/D_-ARs (14), and indicates that these effects are unrelated to AVPR1A-mediated downstream signaling events.

Exposure of cells to both aVP and conivaptan, however, resulted in a reduction in AVPR1A:CR heteromer expression. The observations that aVP reduced the cell surface expression of AVPR1A and its CR partners, and thus reduced the expression of AVPR1A:CR heteromers, are consistent with the previous observation that activation of AVPR1A by aVP results in co-internalization of AVPR1A with its CR heteromerization partner ACKR3 in human vascular smooth muscle cells (19) and the assumption that CXCR1 is not a heteromerization partner of AVPR1A.

The finding that conivaptan does not affect the expression of AVPR1A or CRs but reduces the expression of AVPR1A:CR heteromers mimics the previously described effects of phentolamine on heteromerization between α_1B/D_-AR and their CR partners (15). Because we showed previously that agonist and antagonist binding to α_1B/D_-AR reduce the propensity of α_1B/D_-AR to form heteromers with their CR partners (15), it appears likely that ligand binding to AVPR1A also reduces the heteromerization propensity of AVPR1A for its CR partners, leading to the reduced cell surface expression of AVPR1A:CR heteromers. In combination with our observations after siRNA knockdown in THP-1 cells and in the newly generated AVPR1A^*KO*^ cell line, these data suggest that the formation of heteromeric complexes between CRs with AVPR1A per se regulates the function of the CR partners.

As we observed previously for the CR heteromerization partners of α_1B/D_-ARs (14, 15), heteromerization of AVPR1A with CCR2 or CXCR4 also enhanced the efficacy of the CRs to mediate chemotaxis. Unlike α_1B/D_-ARs within α_1B/D_-AR:CCR1 heteromers, however, heteromerization of AVPR1A with CCR1 reduced the efficacy of CCR1 to mediate chemotaxis, indicating that AVPR1A within AVPR1A:CR heteromers differentially regulates the function of its CR partners.

Moreover, the observed reorganization of the receptor heteromers in ADRA1B^*KO*^ and AVPR1A^*KO*^ cells suggests that α_1B/D_-ARs, AVPR1A, and their shared CR partners exist within interdependent networks of receptor–receptor interactions.

We reported previously that CCR2 and CXCR4 form heteromeric complexes with the α_1B/D_-AR heterodimer, whereas CCR1 heteromerizes with α_1B_-AR and α_1D_-AR protomers or homodimers, but not with the α_1B/D_-AR heterodimer (14). Furthermore, we provided evidence that recombinant and endogenously expressed AVPR1A in human vascular smooth muscle cells heteromerizes with α_1A/B/D_-ARs (9, 13). Thus, it appears likely that CRs, such as CCR2 or CXCR4, form hetero-oligomers with the α_1B/D_-AR heterodimer and AVPR1A and that such hetero-oligomers disassemble if one of the receptor partners is absent. Such a behavior would explain our finding that the absence of AVPR1A in AVPR1A^*KO*^ cells, the absence of the α_1B/D_-AR heterodimer in ADRA1B^*KO*^ cells, and ligand binding to AVPR1A or α_1B/D_-ARs, which interferes with heteromerization, have similar inhibitory effects on CCR2 and CXCR4 function.

The finding that lack of AVPR1A reduces α_1B/D_-AR heterodimerization and increases CCR1:α_1B_-AR and CCR1:α_1D_-AR heteromers at constant expression levels of the individual receptors is consistent with the notion that CCR1 does not interact with the α_1B/D_-AR heterodimer and could be attributed to an increased proportion of α_1B_-AR and α_1D_-AR protomers that are available for heteromerization with CCR1. Moreover, the reduction in CCR1:AVPR1A heteromers in ADRA1B^*KO*^ cells is also consistent with the formation of hetero-oligomeric receptor complexes between CCR1, AVPR1A, and α_1B_-AR or between CCR1, AVPR1A, and α_1D_-AR.

Although the lack of effect of phenylephrine on CCR2-mediated chemotaxis in AVPR1A^*KO*^ cells and of aVP on CCR1- and CCR2-mediated chemotaxis in ADRA1B^*KO*^ cells should be interpreted with caution because of the significantly reduced or absent chemotactic responses in these cells, it would be consistent with the a significant reduction in CCR2:α_1B/D_-AR heteromers in AVPR1A^*KO*^ cells, and in CCR1:AVPR1A and CCR2:AVPR1A heteromers in ADRA1B^*KO*^ cells, respectively. Although the mechanisms leading to the lack of effect of phenylephrine on CCR1-mediated chemotaxis in AVPR1A^KO^ cells, despite increased CCR1:α_1B/D_-AR heteromers in AVPR1A^KO^ cells and increased chemotaxis toward CCL23 of AVPR1A^KO^ cells, remain to be determined, these findings indicate that the presence of AVPR1A is required for the regulatory effects of α_1B/D_-ARs on CCR1 function. This assumption is further supported by our measurements of CCR1-mediated G protein activation in the presence and absence of AVPR1A, α_1B_-AR, and their agonists in a recombinant system, which concurs with our observations in THP-1 cells and monocytes and implies that effects of aVP and phenylephrine on CCR1 function depend on a hetero-oligomeric receptor complex composed of all three receptor partners.

In conclusion, our findings identify AVPR1A as another receptor that heteromerizes with many CRs and controls the function of its CR partners in THP-1 cells and human monocytes. Although methodologies to directly visualize endogenously expressed higher order hetero-oligomeric receptor complexes are currently not available, we provide multiple layers of evidence, suggesting that CRs that share the propensity to heteromerize with α_1B/D_-ARs and AVPR1A exist and function within higher order hetero-oligomeric receptor complexes in cells. Such hetero-oligomeric receptor complexes appear to form interdependent networks of receptor–receptor interactions through which ligand-free and ligand-bound α_1B/D_-ARs and AVPR1A allosterically modulate the efficacy of the CR partners to mediate chemotaxis. Consistent with our observations on CCR1-mediated Gαi activation in the presence of ligand-bound α_1B_-AR and AVPR1A, such effects could be explained by conformational re-arrangements of the CR partners upon heteromerization with α_1B/D_-ARs and AVPR1A, which affect with efficacy of the CR partners to activate Gαi. To gain initial insights into the possible structural determinants underlying the observed effects of α_1B_-AR and AVPR1A and to dissect the potential mechanisms that enable AVPR1A to differentially regulate CR function, detailed molecular dynamics simulations of hetero-oligomeric receptor complexes may be useful to generate testable hypotheses, which could then be validated in mutagenesis experiments in the future.

Similar to the IC_50_ of α_1_-AR ligands to inhibit chemotaxis mediated by CR partners of α_1B/D_-ARs (14), the EC_50_ and IC_50_ of AVPR1A ligands to modulate the efficacy of CR partners of AVPR1A are in the range of physiologically and pharmacologically relevant concentrations (26, 27, 28), which implies functional relevance of our findings in health and disease processes. Although it is known since decades that catecholamines influence leukocyte trafficking in health and disease and that activation of AVPR1A increases leukocyte counts in the systemic circulation, the underlying molecular mechanisms are unknown (29, 30, 31). Because chemokines and their receptors are recognized as the most critical regulators of leukocyte positioning and margination, our findings provide a molecular mechanism for such phenomena and suggest that hetero-oligomeric complexes composed of CRs, α_1B/D_-ARs, and AVPR1A may constitute the molecular basis through which stress regulates innate immune cell trafficking and positioning.

## Materials and Methods

### Antibodies, proteins, and reagents

The antibodies anti-α_1B_-AR (host: rabbit; catalog#: ab169523), anti-α_1D_-AR (host: rabbit; catalog#: ab84402), and anti-CXCR4 (host: goat, ab1670) were obtained from Abcam; anti-AVPR1A (host: rabbit; catalog#: bs-11598R) was from Bioss; anti-AVPR1A (host: mouse, catalog#: LS-C126889) and anti-CCR8 (host: goat; catalog#: LS-C187704) were from LifeSpan Biosciences; anti-CXCR1 (host: rabbit, catalog#: PA5-33452) was from Invitrogen; and anti-CCR1 (host: mouse; catalog#: MAB145), anti-CCR2 (host: mouse; catalog#: MAB48607), IgG isotype control (host: rabbit, catalog#: MAB1050), IgG isotype control (host: mouse, catalog#: MAB004), and IgG isotype control (host: goat, catalog#: AB-108-C) were from R&D Systems. CCL2, CCL23_25-99_, CXCL8, and CXCL12 were purchased from Protein Foundry. Phenylephrine, phentolamine, arginine vasopressin, conivaptan, and poly-L-lysine were purchased from Sigma-Aldrich. AVPR1A siRNA and nontargeting (NT) siRNA and Accell transfection media were purchased from GE Dharmacon. Proximity ligation rabbit, mouse, and goat +/− probes and detection reagents were purchased from Sigma-Aldrich.

### Plasmids

cDNA for all 23 CRs was obtained from Addgene, except for cDNAs encoding CCR1, CCR9, XCR1, ACKR1, ACKR2, and ACKR5, which were bought from the Arizona State University. cDNAs encoding metabotropic glutamate receptor 1 (mGlu_1_R), α_1b_-AR, and AVPR1A were also purchased from Addgene. BRET sensor, GPCR-Rluc or GPCR-YFP, was produced by inserting DNAs encoding EYFP or Renilla luciferase (Rluc) in frame at the C-terminus of the above GPCRs into the Age I/Xba I sites, using IDTG (single-letter amino acid code) as spacer sequence between the GPCR C-termini and the BRET sensors. TRUPATH biosensors, Gαi1-Rluc8, Gβ3, and Gγ9-GFP2, were from Addgene deposited by the laboratory of Dr. Bryan Roth (32). All plasmid sequences were verified using Sanger sequencing.

### Cells and cell lines

The human monocytic leukemia cell line THP-1 was from the American Type Culture Collection (ATCC) and cultured as previously described (14, 15). Briefly, cells were cultured and maintained in RPMI 1640 (Sigma-Aldrich) supplemented with 10% FBS, 100 U/ml penicillin, and 100 μg/ml streptomycin (Invitrogen). HEK293T cells were obtained from the ATCC and maintained in DMEM (Sigma-Aldrich) supplemented with 10% FBS, 100 U/ml penicillin, and 100 μg/ml streptomycin. ADRA1B^*KO*^ cells, a CRISPR/Cas9 gene-edited THP-1 cell line that lacks ADRA1B, were cultured as described previously (14, 15).

Human monocytes were isolated from whole blood from healthy volunteers, according to our institutional review board (IRB) protocol approved by the University of South Florida. Informed consent was obtained from all individual participants included in the study. Whole blood was drawn by venipuncture into sodium citrate CPT mononuclear cell preparation tubes from Becton Dickinson, and peripheral blood mononuclear cells were isolated by density gradient centrifugation at 1,800*g* for 20 min. CD14+/CD16− monocytes were then isolated via negative selection using magnetic-activated cell sorting LS columns (MACS LS), an indirect magnetic labeling system from Miltenyi Biotec. MACS LS uses a mixture of biotin-conjugated monoclonal anti-human antibodies against CD3, CD7, CD16, CD19, CD56, CD123, and CD235a for negative selection (catalog#: 130-117-337). The purity and composition of the monocyte preparations were assessed by morphology and by measuring the cell size, granularity, and expression of CD14/CD16 by flow cytometry.

### Gene silencing by RNA interference

Gene silencing with siRNA was performed as described previously (14, 15). In brief, to deplete AVPR1A from the cell surface, cells were incubated in Nunc six-well plates (Thermo Fisher Scientific) for 3 d in Accell transfection media (GE Dharmacon) with AVPR1A or NT (negative control) siRNA (GE Dharmacon) at a concentration of 1 μM. On day 3, cells were centrifuged at 300*g* for 5 min and resuspended in RPMI 1640 (Sigma-Aldrich) supplemented with 10% HyClone FBS from Cytiva, 100 μg/ml penicillin from Invitrogen (Waltham, MA), and 100 μg/ml streptomycin (Invitrogen). Cells were used on day 4 for experimentation.

### CRISPR/Cas9 gene editing

CRISPR/Cas9 gene editing was performed as previously described (14). To generate the lentivirus encoding both Cas9 and sgRNA targeting AVPR1A (target sequence ACAGCCGACCGCTACATCG), 293T cells were co-transfected with the LV01 lentivirus plasmid (synthesized by Sigma-Aldrich) and lentiviral packaging mix (Sigma-Aldrich) using Lipofectamine 2000 (Invitrogen). After overnight incubation, the culture medium was replaced with THP-1 medium RPMI 1640. 2 d post-transfection, the supernatants containing the lentiviral particles were collected and spun at 500*g* for 5 min. The resultant supernatant was filtered and used to transduce THP-1 cells. 2 d after transduction, cells were selected by the addition of puromycin (1 μg/ml). To check the efficiency of CRISPR, the genomic DNA from transduced cells was extracted with DNAzol (Invitrogen). The DNA sequence flanking the targeting region was amplified by PCR with primers (forward: CCG CAT GCA CCT CTT CAT, reward: AAA TGG ACT TCA CGC TGC T) using Platinum Blue PCR SuperMix (Invitrogen) according to the manufacturer’s instructions. The PCR product was examined for mutations with the T7EI assay kit (Integrated DNA Technologies) following the manufacturer’s instructions. After confirming efficient editing of AVPR1A genomic DNA, the transduced cells were replated in 96-well plates at 1 cell/well in the conditioned medium containing 20% FBS. 3 wk later, clones were replated in 24-well plates and subjected to screening with the T7EI assay kit. To detect the sequences of both alleles, DNAs from the edited clones were PCR-amplified with the above primers and subcloned to the TA cloning vector. The resulting plasmids were sequenced. The clones containing out-of-frame inserts or deletions in both alleles (designated AVPR1A^*KO*^) were expanded for experiments.

### BRET screening and saturation BRET

Assays were performed as previously described (14). Briefly, HEK293T cells were transfected using Lipofectamine 3000 (Thermo Fisher Scientific). For screening assays, 5 ng of AVPR1A-Rluc was transfected with 25 ng of CR-YFP or increasing amounts of the mGlu_1_R-YFP (ctrl.). For saturation BRET experiments, 5 ng of AVPR1A-Rluc was transfected with increasing amounts of CR-YFP. For each experiment, empty vector pcDNA3.1 was added to keep the total amount of DNA for each transfection constant. After transfection, cells were incubated overnight at 37°C in a humidifying chamber and then replated to poly-L-lysine–coated 96-well plates (Greiner Bio-One). BRET assays were performed when replated cells reached ≥80% confluence. Fluorescence was measured with a plate reader (Cytation 1 Cell Imaging Multi-Mode Reader; BioTek) (excitation 485 nm, emission 528 nm). For BRET measurements, coelenterazine H was used at a final concentration of 5 μm as the luminescent substrate. Luminescence was measured at 460 and 528 nm. The BRET signal was calculated as the ratio of the relative luminescence units (RLUs) measured at 528 nm to RLUs at 460 nm. The net BRET was calculated by subtracting the BRET signal detected when AVPR1A-Rluc was transfected alone. For screening and saturation experiments, net BRET ratios are expressed as a function of fluorescence/total luminescence.

### PLA

Cells (THP-1 cells, CRISPR/Cas9 knockout clones, siRNA-pretreated cells) were deposited in monolayers on glass slides (Thermo Fisher Scientific) by centrifugation at 800*g* using Cytospin 4 Centrifuge (Thermo Fisher Scientific). Cell monolayers were generated with a seeding volume of 200 μl at a seeding density of 100 cells/μl, totaling 20,000 cells per funnel (Thermo Fisher Scientific). Cell monolayers were subsequently isolated into individual wells using a water-repellent solution (Super PAP Pen; Thermo Fisher Scientific). Deposited cells were fixed with 4% (wt/vol) paraformaldehyde for 15 min at RT and then blocked overnight at 4°C with Sigma-Aldrich Duolink PLA blocking reagent. Blocked slides were incubated with indicated primary antibody(s) in dilutions of 1 μg/ml corresponding to the receptor(s) of interest. IgG isotype antibodies were used as a control. Slides were subsequently washed with PBS and incubated for 60 min at 37°C in a humidifying chamber, room air with secondary species‐specific antibodies conjugated to plus and minus PLA probes (1:5). Probed slides were then washed with Sigma-Aldrich Duolink wash buffer A and incubated with ligation reagent (30 min at 37°C in a humidifying chamber, room air). Slides were then washed again with wash buffer A and incubated with amplification reagent (105 min at 37°C in a humidifying chamber, room air). Amplified slides were washed twice with wash buffer B and then once with 0.01× wash buffer B and allowed to dry. Treated slides were mounted with 50 μl per well of Duolink in situ mounting medium with DAPI overnight at -20°C. PLA signals (Duolink in situ detection reagents red [λ excitation/emission 598/634 nm]) were identified as fluorescent hotspots under a Keyence BZ-X710 fluorescence microscope (60×/1.50 oil) at RT from merged z-stack (thickness 0.5 μm, bottom to top) images. PLA signals were quantified using Fiji, an open source image processing package based on ImageJ2 (National Institute of General Medical Sciences). Images were imported in merged.tiff formats containing both signal and nucleus channels. Merged images were visually verified for analytical quality. Comparisons and statistical analyses were performed only when PLAs were performed on the same day in parallel experiments. Fluorescence microscopy was performed with identical settings. For each experiment and condition, 10 randomly selected nonoverlapping vision fields were analyzed.

### Chemotaxis assays

Cellular migration was measured employing the Neuroprobe ChemoTx Disposable Chemotaxis System. A 96-well Boyden chamber (30 μl/well, 8 μm pores, 1 × 10^5^ pores/cm^2^ pore density) was selected for all chemotaxis experiments. The bottom wells of the Boyden chamber were loaded with 30 μl of test substances at various concentrations. The top wells of the membrane were loaded with 25 μl of cells (THP-1 cells, CRISPR/Cas9 knockout cell lines, siRNA-pretreated cells, or freshly isolated monocytes) (200 cells/μl). THP-1 cells and their derivatives were suspended in depleted RPMI 1640 (0.5% FBS, Cytiva); freshly isolated monocytes were suspended in RPMI 1640 (0.5% human platelet-poor plasma specific to the sample donor). After 3 h, the membrane was carefully removed and transmigrated cells were transferred from the bottom wells of the 96-well Boyden chamber to a Nunc, clear, flat-bottom 96-well plate (Thermo Fisher Scientific). The plate was covered, and transferred cells were allowed to settle to the bottom of the plate for 30 min. Transferred cells were counted using the Cytation 1 plate reader (BioTek) by direct imaging in a high-contrast bright field (4x) and post-imaging particle analyses with Gen5 (v3.05) Imaging & Microscopy Software (BioTek). The chemotactic index (CI) was calculated as the ratio of cells that transmigrated in the presence versus the absence of the test solutions.

### Gαi activation assays

Gαi activation was measured using the TRUPATH biosensor method (22, 32, 33). HEK293T cells were plated in a six-well plate and transfected with 0.2 μg each of Gαi1-Rluc8, Gβ3, and Gγ9-GFP2 (TRUPATH) together with 0.1 μg CCR1 with either α_1b_-AR, AVPR1A, both, or empty vector pcDNA3.1 as a negative control. 24 h after transfection, cells were trypsinized and replated to 96-well poly-L-lysine–precoated plates. After overnight incubation, cells were replaced with 0.1% glucose/PBS. Coelenterazine 400a in a final concentration of 5 μM was added as the luminescent substrate and incubated at RT for 3 min. Ligands at various concentrations were added to cells and incubated at RT for 3 min before luminescence was measured at 410 and 515 nm. The BRET signal was calculated as the ratio of the relative luminescence units (RLUs) measured at 515 nm to RLUs measured at 410 nm. The BRET changes were calculated by subtracting the BRET signal of untreated cells.

### Data analyses and statistics

Data are expressed as the mean ± SE of the mean from n independent experiments that were performed on different days. Data were analyzed using GraphPad Prism v. 9.5.1 software. BRET and dose–response curves were analyzed using nonlinear regression analyses. *t* test, one-way, and two-way ANOVA with Dunnett’s multiple comparisons test were used as appropriate. A two-tailed *P* < 0.05 was considered significant.

## Supplementary Material

Reviewer comments

## Data Availability

All datasets are included in the study. This study was performed in line with the principles of the Declaration of Helsinki. Approval was granted by the Ethics Committee of the University of South Florida (Pro00037030/Date 07/13/2023). Informed consent was obtained from all individual participants included in the study. Consent to publish has been received from all participants.
